# Acrometastasis as an Initial Sign of Progression in Untreated Metastatic Renal Cell Carcinoma: A Case Report

**DOI:** 10.7759/cureus.112982

**Published:** 2026-07-19

**Authors:** Lynnette RL Tan, Tina Munn Yi Lee, Puey Ling Chia, Yee Lin Tang, Zhen Wei Choo

**Affiliations:** 1 Urology, Tan Tock Seng Hospital, Singapore, SGP; 2 Hand and Reconstructive Microsurgery, Tan Tock Seng Hospital, Singapore, SGP; 3 Medical Oncology, Tan Tock Seng Hospital, Singapore, SGP; 4 Pathology, Tan Tock Seng Hospital, Singapore, SGP

**Keywords:** acrometastasis, bone neoplasms, clear cell carcinoma, hand surgery, renal cell carcinoma

## Abstract

Acral metastases are an exceptionally uncommon manifestation of bone dissemination, with renal cell carcinoma (RCC) serving as a particularly rare primary source. Historically, these lesions signified terminal disease managed with palliative amputation, although most data predate modern immunotherapy.

A man in his 50s presented with newly diagnosed clear cell RCC, an inferior vena cava tumor thrombus, and bilateral lung metastases. He repeatedly declined systemic immunotherapy. Nine months later, he developed a painful swelling of the right ring fingertip. Imaging revealed an osteolytic lesion of the distal phalanx. Amputation was performed, and histopathology confirmed metastatic RCC.

This case illustrates an atypical acral metastasis secondary to untreated, naturally progressing systemic disease. It highlights the challenges of patient autonomy and traditional medicine preferences. It emphasizes the role of local surgery for symptom relief and histological verification and underscores the necessity of a highly individualized, multidisciplinary approach to managing rare metastatic presentations.

## Introduction

Acrometastasis, the occurrence of metastases distal to the elbows or knees, accounts for approximately 0.1% of skeletal spread. Among these rare lesions, renal cell carcinoma (RCC) represents about 10%-12%, second only to lung carcinoma [1]. Historically, such lesions have implied widespread disease and poor prognosis, with a median survival of six months, often leading to amputation for palliation.

The clinical landscape of metastatic RCC has changed dramatically. Immune checkpoint inhibitors (ICI) combined with tyrosine kinase inhibitors (TKI) have redefined survival outcomes and functional expectations, shifting management goals from palliation to durable control. However, existing acrometastasis literature largely predates these advances.

This report describes digital metastasis developing in a patient with radiologically confirmed metastatic clear cell RCC who deferred systemic therapy, followed by amputation and subsequent reassessment for ICI initiation.

## Case presentation

**Figure 1:**
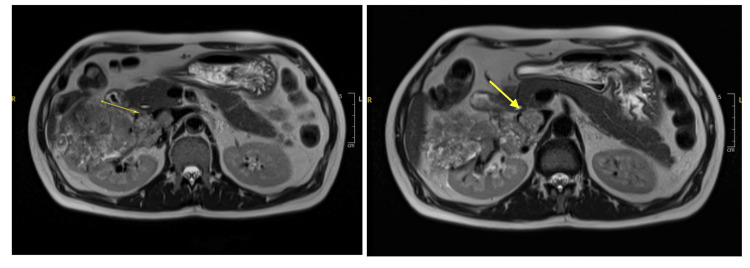
Axial views of MRI kidneys showing a locally advanced right renal mass with tumor thrombus in the right renal vein (left, arrow) and tumor thrombus in the infrahepatic IVC (right, arrow) MRI: magnetic resonance imaging; IVC: inferior vena cava

**Figure 2:**
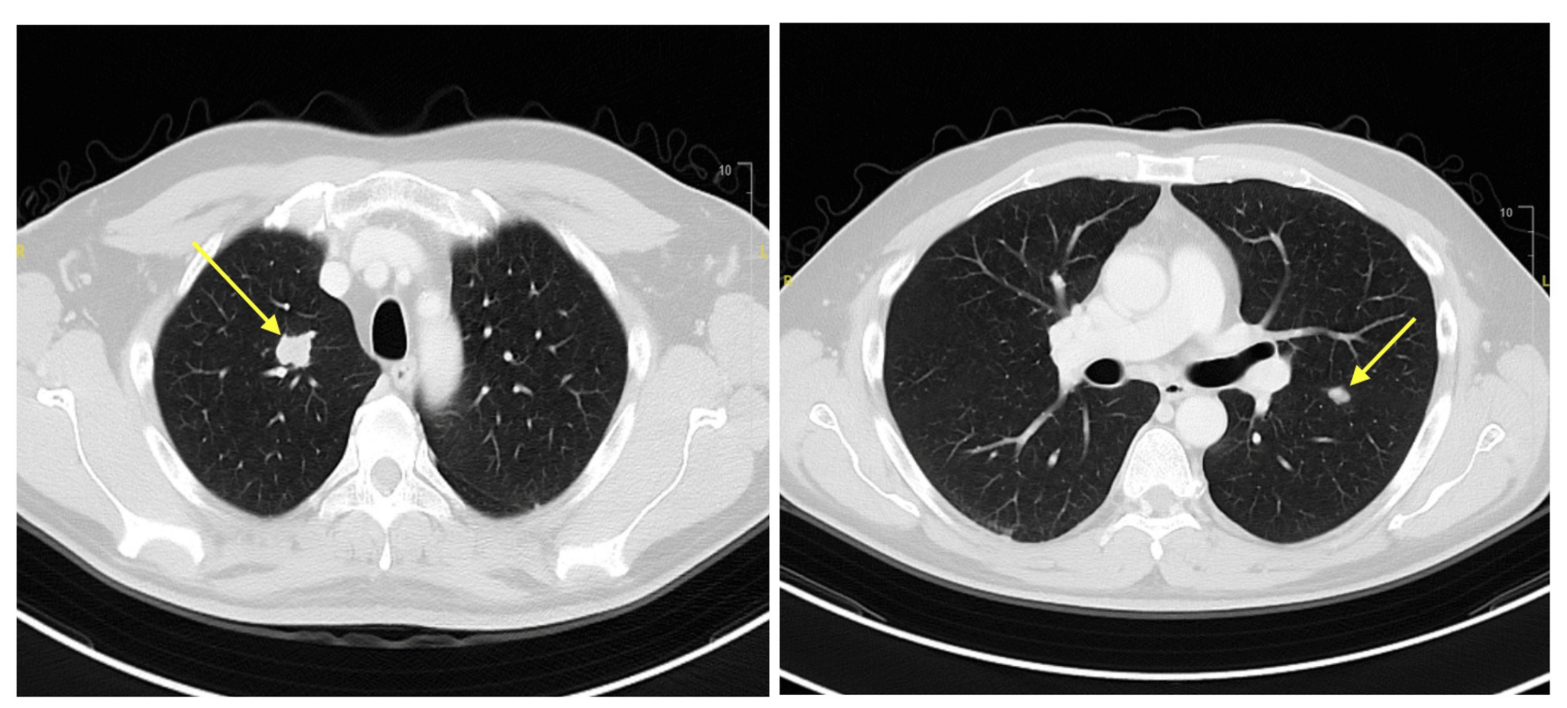
Axial view of CT thorax showing bilateral pulmonary nodules suggestive of metastatic disease (arrows) CT: computed tomography

A male in his 50s, with a 30-year smoking history and recently diagnosed type 2 diabetes mellitus, presented with visible hematuria. Computed tomography (CT) urography and magnetic resonance imaging (MRI) of the kidneys revealed an 8.5 × 7.4 cm right mid- to lower-pole renal mass invading the renal pelvis and extending as a tumor thrombus into the renal vein (Figure 1) and infrahepatic inferior vena cava (IVC) (Figure 1). The tumor abutted the ascending colon without definite invasion. CT thorax demonstrated multiple bilateral pulmonary nodules, the largest measuring 1.7 cm in the right upper lobe (Figure 2). Radiologically, these were considered metastatic from RCC, although biopsy was declined. The findings corresponded to clinical stage IV (cT3b N0 M1) clear-cell RCC. After multidisciplinary counseling, the patient declined systemic therapy and pursued traditional medicine.

Follow-up MRI of the kidneys showed a stable renal mass and unchanged IVC thrombus. Interval CT thorax confirmed progression in the size and number of pulmonary metastases. Concurrently, the patient noted a gradually enlarging, tender swelling at the right ring fingertip over two months, without antecedent trauma or infection. He was seen by a hand surgery specialist, where examination revealed bulbous expansion of the distal phalanx, with erythema, nail plate lifting, and ulceration.

**Figure 3:**
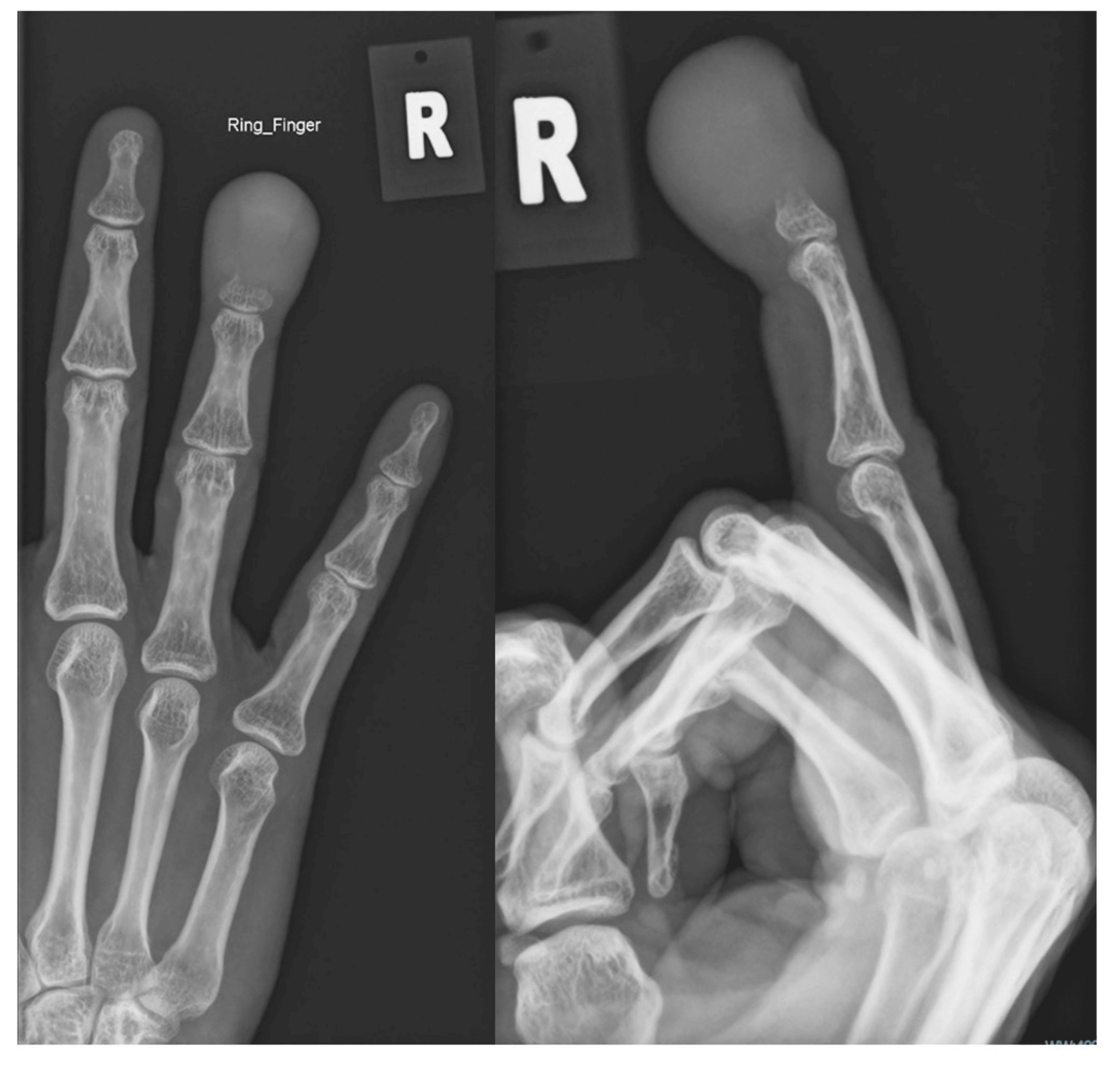
X-ray of patient's right ring finger in A/P (left) and lateral (right) views Both images demonstrate a soft tissue mass in the pulp of the distal phalanx of the right ring finger, with erosion of underlying bone.

Plain radiographs showed a soft-tissue mass with osteolytic erosion of the right ring finger distal phalanx (Figure 3). 

Given increasing pain and ulceration, distal phalanx amputation was performed. Intraoperatively, there was a friable, exophytic mass destroying the distal phalanx, confined distally by healthy bone proximally.

**Figure 4:**
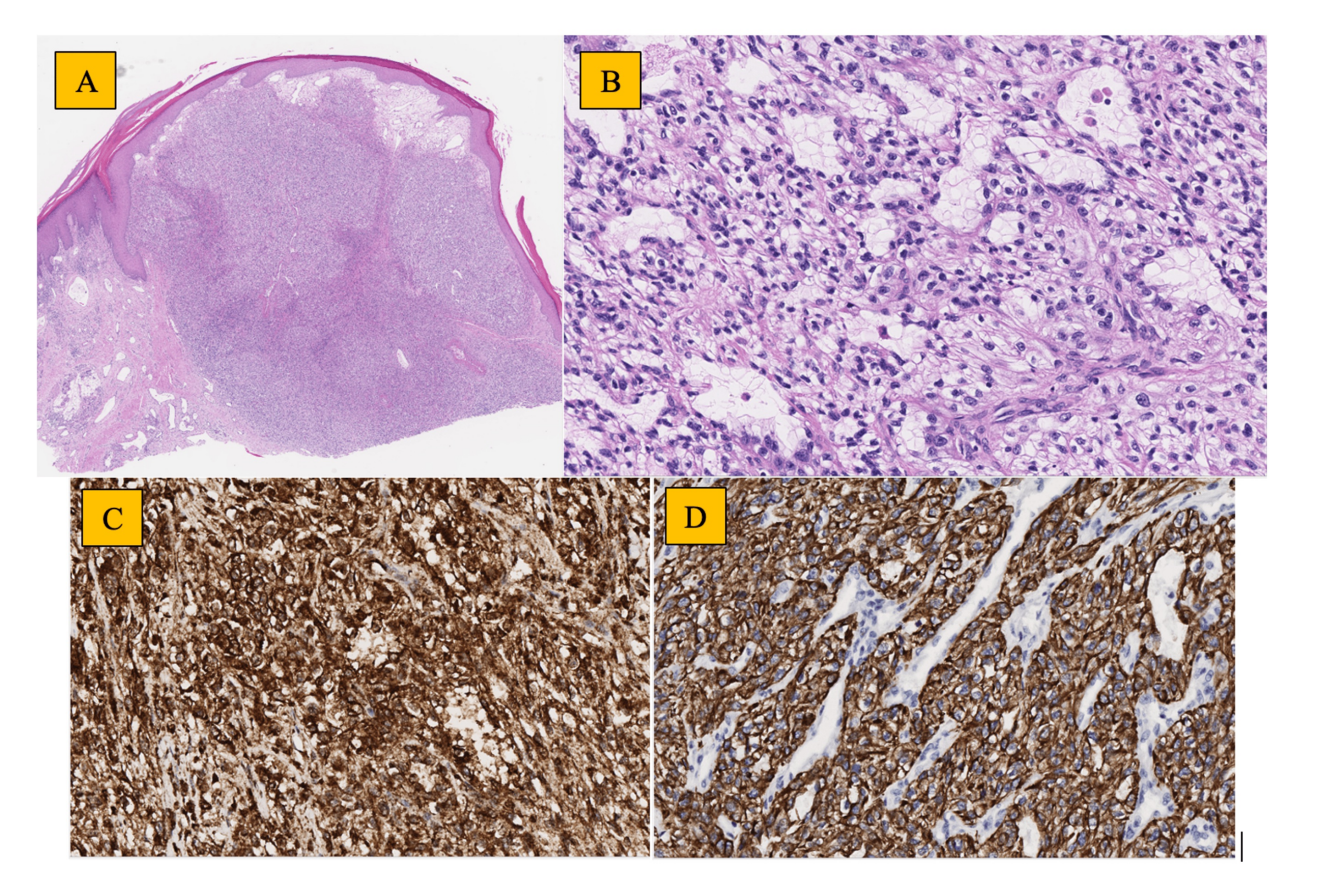
Histopathological examination of right ring finger tumor A (H&E, ×1.2): Tumor in the skin and subcutaneous tissue of the finger. B (H&E, ×20): Nested appearance of tumor cells surrounded by fibrovascular septa. The tumor cells have well-defined cell borders and clear cytoplasm. C (×20): Tumor cells diffusely positive for RCC-Ma. D (×20): Tumor cells diffusely positive for AE1/3.

Microscopy revealed epithelioid-to-spindle tumor cells forming nests and clusters within fibrovascular septa, showing moderate pleomorphism and clear cytoplasm. Immunohistochemistry was positive for AE1/3, RCC-Ma, and CD10 and negative for CK7, CK20, CDX2, TTF-1, Melan-A, and HMB-45, confirming metastatic clear cell RCC. Margins were clear, with the closest soft-tissue margin being 0.6 cm (Figure 4).

Recovery was uneventful. Subsequent imaging demonstrated stable renal and pulmonary disease. The patient initially agreed to consider systemic immunotherapy; however, following renewed counseling by medical oncology, he again opted against treatment.

## Discussion

Diagnostic challenges

Acrometastasis is rare, representing only 0.1% of all bone metastases. These lesions frequently present with nonspecific symptoms such as erythema, bulbous swelling, and ulceration, often leading to diagnostic mimicry of chronic paronychia, arthritis, or osteomyelitis [2,3]. Hence, clinically, it may be difficult to distinguish benign from malignant lesions, especially in patients with no history of underlying malignancy. Nevertheless, the index of suspicion should be heightened in patients with underlying malignancy, as it can be a sign of distant metastasis, commonly from the lung, with kidney cancer a close second [1]. The need for histological verification is essential. Our case reinforces this need, given that these lesions may also be a sign of systemic progression [4]. 

Clear cell RCC at acral sites

While acrometastasis is an uncommon manifestation of clear cell RCC, its development aligns with the known hematogenous propensity of this malignancy rather than a distinct pathogenic mechanism. The route of acral dissemination typically depends on the primary site: subdiaphragmatic tumors may utilize the valveless Batson’s plexus to reach the lower limbs, whereas supradiaphragmatic tumors or those with systemic arterial dissemination reach the upper extremities via the pulmonary circulation. Although the rapid, high-pressure capillary network of peripheral digits is generally hostile to tumor emboli, acrometastasis frequently correlates with multi-organ involvement. Notably, two-thirds of patients with digital metastasis present with concurrent secondary lesions elsewhere, most frequently in the lungs [5,6]. In our patient, the absence of intervening axial skeletal involvement makes isolated Batson’s plexus dissemination unlikely. Instead, the interval progression of pulmonary metastases closely following the appearance of the acral lesion strongly supports a sequential cascade wherein tumor emboli first seeded the lungs before ultimately gaining access to the systemic arterial circulation to reach the distal phalanx.

Histologic confirmation enabling systemic therapy initiation

In our case, surgical intent was more than just simple pain relief or infection control; it included histological verification, as the patient refused lung biopsy, as well as tumor burden reduction and functional restoration. This stabilized the patient and allowed an opportunity to initiate immunotherapy that might not have otherwise occurred. Medical oncologists can also more accurately assess the efficacy of ICI and TKI combinations on the remaining systemic disease burden [7].

Patient autonomy and continuity of care

In Singapore's multicultural healthcare landscape, up to 50% of adult cancer patients utilize complementary and alternative medicine [8]. In this case, respecting the patient’s initial decision to defer standard therapy while maintaining rigorous active surveillance was critical. Rather than discharging him, adhering to a strategy of continuous clinical engagement preserved the patient-physician relationship. This continuity was vital: it ensured the patient remained within the tertiary care network, allowing for the timely detection of disease progression via interval imaging and rapid multidisciplinary coordination between the hand surgery team and the tumor board following the finger amputation. Ultimately, this proactive engagement facilitated a swift re-referral and successful transition back to systemic oncological therapy.

## Conclusions

Acrometastasis is a rare indicator of systemic progression and hematogenous dissemination, even when the primary tumor remains radiologically stable. In patients who defer treatment, maintaining continuous clinical engagement is vital; it respects autonomy while ensuring timely recognition of disease progression and re-referral for systemic therapy. For digital lesions, surgical amputation serves a dual diagnostic and therapeutic role, providing definitive histology and local disease control. Larger prospective studies remain necessary to better understand long-term oncological outcomes.
